# Intrinsic and Extrinsic Properties Affecting Innate Immune Responses to Nanoparticles: The Case of Cerium Oxide

**DOI:** 10.3389/fimmu.2017.00970

**Published:** 2017-08-14

**Authors:** Eudald Casals, Muriel F. Gusta, Jordi Piella, Gregori Casals, Wladimiro Jiménez, Victor Puntes

**Affiliations:** ^1^Vall d’Hebron Institut of Research (VHIR), Barcelona, Spain; ^2^Institut Català de Nanociència i Nanotecnologia (ICN2), CSIC and The Barcelona Institute of Science and Technology (BIST), Campus UAB, Barcelona, Spain; ^3^Biochemistry and Molecular Genetics Service, Hospital Clínic Universitari, IDIBAPS, CIBERehd, Barcelona, Spain; ^4^Department of Biomedicine, University of Barcelona, Barcelona, Spain; ^5^Institució Catalana de Recerca i Estudis Avançats (ICREA), Barcelona, Spain

**Keywords:** nanoparticles, cerium oxide, nanoparticle evolution, nanoparticle agglomeration, ion leaching, antioxidant activity, inflammation, immune response

## Abstract

We review the apparent discrepancies between studies that report anti-inflammatory effects of cerium oxide nanoparticles (CeO_2_ NPs) through their reactive oxygen species-chelating properties and immunological studies highlighting their toxicity. We observe that several underappreciated parameters, such as aggregation size and degree of impurity, are critical determinants that need to be carefully addressed to better understand the NP biological effects in order to unleash their potential clinical benefits. This is because NPs can evolve toward different states, depending on the environment where they have been dispersed and how they have been dispersed. As a consequence, final characteristics of NPs can be very different from what was initially designed and produced in the laboratory. Thus, aggregation, corrosion, and interaction with extracellular matrix proteins critically modify NP features and fate. These modifications depend to a large extent on the characteristics of the biological media in which the NPs are dispersed. As a consequence, when reviewing the scientific literature, it seems that the aggregation state of NPs, which depends on the characteristics of the dispersing media, may be more significant than the composition or original size of the NPs. In this work, we focus on CeO_2_ NPs, which are reported sometimes to be protective and anti-inflammatory, and sometimes toxic and pro-inflammatory.

## Introduction

Nanotechnology has already qualified as the industrial revolution of the twenty-first century. Although its development is a logical continuation of the development of microelectronics and colloid chemistry, the beginning of the *nano era* corresponds, for most people, with Smalley’s synthesis of fullerene (C_60_) (1). Since then, organic nanomaterials (e.g., C_60_, carbon nanotubes, graphene) have garnered much interest, but have also generated concerns regarding toxicity (2–4). Meanwhile, the development of inorganic nanomaterials has caused far less controversy, and it is only in the past few years that some of these materials (e.g., TiO_2_, Ag, Fe_3_O_4_) have come under closer scrutiny to address human and environmental toxicity issues (5–7). It has also become increasingly common to examine the effects of a nanocomposite or nano-enabled products instead of the pristine nanoparticle (NP) alone. Indeed, the effects of the “active ingredient” can be (and actually often are) deeply modified by the formulation of the final product and the properties of the media in which it is dispersed. This highlights the complexity of addressing the fate of a nanomaterial through its life cycle in a meaningful manner.

Cerium oxide nanoparticles (CeO_2_ NPs) have recently received much attention because of their excellent catalytic redox properties (8). In addition to being a rather chemically inert ceramic, a CeO_2_ nanocrystal has a fluorite-like structure where the unfilled 4f electronic orbital confers it a variety of relevant catalytic properties when it reaches the nanoscale. Consequently, nanoceria has been used in the petrochemical industry and in catalytic exhaust converters for decades. CeO_2_ NPs have high capacity to buffer electrons in redox environments due to the ease of oxidation and reduction from Ce^3+^ to Ce^4+^ and *vice versa* (9, 10), followed by the capture or release of oxygen. As a consequence, they act as *electron sponges* in the presence of free radicals degrading thus reactive oxygen species (ROS) (11). In detail, inflammation and oxidative stress are interconnected processes that contribute decisively to the pathogenesis of many diseases, including highly prevalent, age-related disorders, such as obesity, cardiovascular disease, diabetes mellitus, cancer, chronic respiratory diseases, and neurological diseases. Mutual stimulation between oxidative stress and inflammation contributes decisively to the chronic nature of these diseases. Oxidative stress involves elevated intracellular levels of ROS, such as peroxides, superoxides, hydroxyl radicals, and singlet oxygen, which have critical roles in physiological processes through the regulation of cell signaling cascades. Prolonged exposure to high ROS concentrations damages proteins, lipids, and nucleic acids, causing various metabolic complications.

Thus, CeO_2_ NPs in the size range of 3–50 nm have recently received increased attention for their participation in biochemical redox reactions, providing sites for free radical scavenging and reducing inflammation (12–14). Thus, CeO_2_ NPs have been reported to confer cellular protection, especially in the reduction of oxidative and nitrosative stress in living organisms, and are considered an alternative approach offering new opportunities for the treatment of physiopathological processes leading to chronic inflammation (15).

In this regard, most therapeutic CeO_2_ NPs applications are proposed based on their ability to reduce ROS levels and consequently, the levels of most inflammatory mediators, such as inducible nitric oxide synthase, nuclear factor κβ, tumor necrosis factor-α, and interleukins (16–19). Indeed, CeO_2_ NPs were recently found to have multi-enzyme mimetic properties, including those related to superoxide dismutase (SOD), catalase, and oxidase (8). In this context, CeO_2_ NPs have potential applications in many different medical fields. For example, in cardiology, intravenously administered CeO_2_ NPs in a transgenic murine model of cardiomyopathy were proved to reduce the myocardial oxidative stress, the endoplasmic reticulum stress, and suppress the inflammatory process, ensuring protection against progression of cardiac dysfunction (20). In oncology, antioxidant properties of CeO_2_ NPs were successfully tested to protect cells from radiation-induced damage (21). In another study, CRL8798 cells (immortalized normal human breast epithelial cell line) and MCF-7 (a breast carcinoma cell line), were exposed to radiation and CeO_2_ NPs were reported to confer radioprotection to the normal human breast line but not to the tumoral one (22). In hepatology, CeO_2_ NPs were shown to display hepatoprotective effects against steatosis in rats with diet-induced non-alcoholic steatohepatitis (23) and to reduce steatosis, portal pressure, and ameliorate systemic inflammatory biomarkers, attenuating the intensity of the inflammatory response in a model of rats with induced liver fibrosis. In ophtalmology, CeO_2_ NPs are being tested to treat ocular diseases such as macular degeneration and glaucoma. The ability of CeO_2_ NPs to protect retinal neurons was shown for primary cell cultures of dissociated rat retinas injecting the suspension of CeO_2_ NPs into the vitreous of both eyes (9). Similarly, beneficial effects of the use of CeO_2_ NPs have been found in the case of neurodegenerative diseases (24). In this studies, CeO_2_ NPs are shown to display SOD mimetic activity (25, 26), catalase mimetic activity (11, 27), and/or nitric oxide (^⋅^NO) scavenging abilities (17). Last, CeO_2_ NPs are also amenable to local targeting and delivery, as shown in the works of Li et al., (28) and Xu et al (29).

## Positive and Negative Effects of NPs

Obviously, the safe and effective use of these promising therapeutic NPs requires the precise assessment of their potential risks and unwanted side effects. Despite the vast range of publications that address the toxicity and safety of nanomaterials, results are still controversial, with different observed effects for similar NPs ranging from severely toxic effects—as in the study of Kovriznych et al. (30), which assess and compare the acute toxicity of 31 different nanomaterials to fish mature individuals of *Danio rerio*—to innocuous [e.g., Ref. (31)] or beneficial [e.g., Ref. (32, 33)]. CeO_2_ NPs are no exception. While they have been reported many times to be safe and beneficial, protecting against oxidative stress (9, 13, 21, 22, 34), other studies, mainly related to the toxicity of CeO_2_ nanopowders employed in industry, reported *in vitro* and *in vivo* toxicity (35, 36). In addition, while some studies report CeO_2_ NP uptake by hepatocytes and anti-inflammatory effects in the liver (14, 37), others report macrophage (Kupffer cell) uptake and pro-inflammatory effects (38).

**Figure 1:**
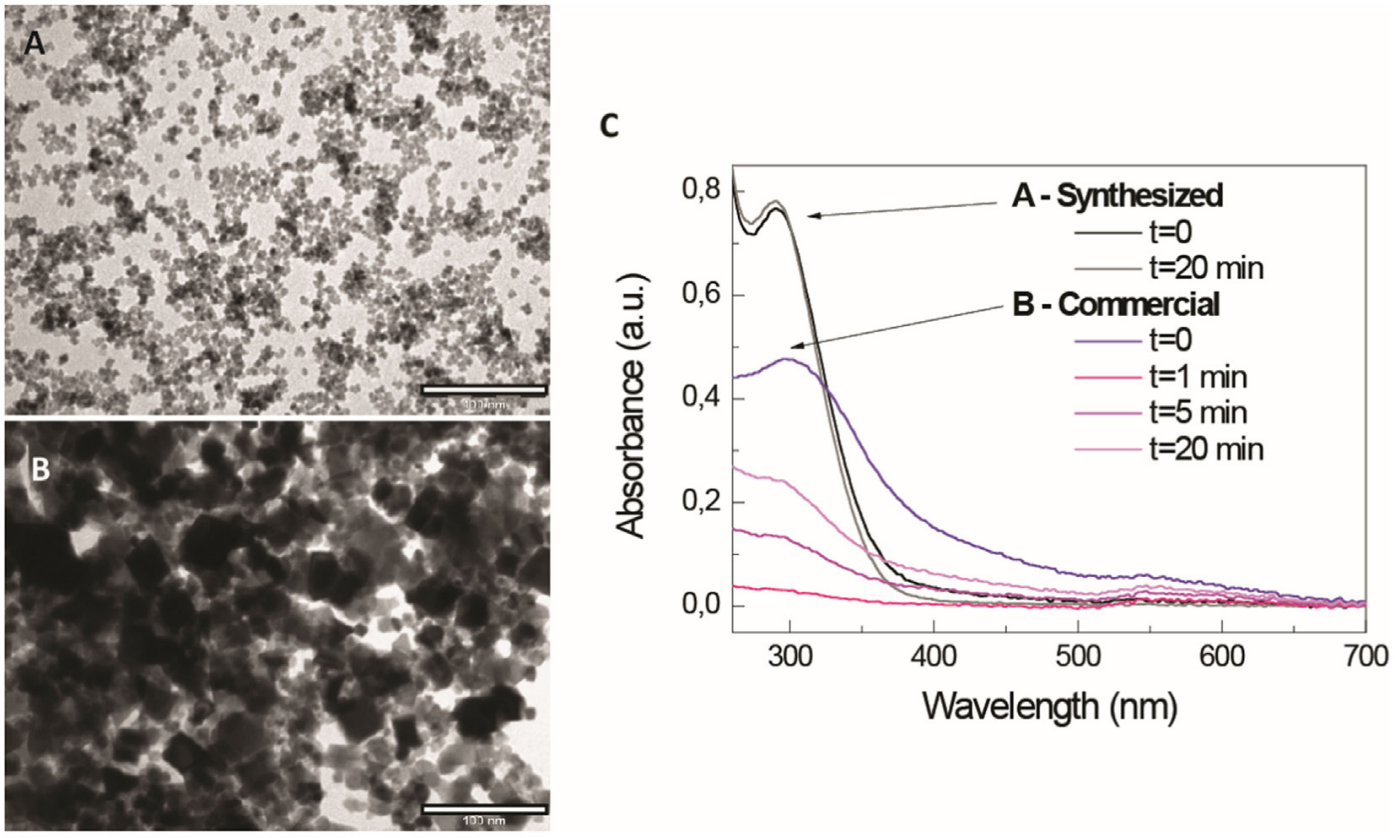
Different aspect and stability of commercial and designed CeO_2_ nanoparticles (NPs). Different morphologies and sedimentation behavior of CeO_2_ nanopowders (commercial, nominal size <25 nm) and CeO_2_ NPs synthesized in the laboratory after dispersion in TMAOH 1 mM, a good stabilizer of metal oxide NPs. **(A,B)** Representative TEM images CeO_2_ NPs and CeO_2_ nanopowders, respectively (scale bar = 100 nm); **(C)** UV-VIS spectroscopy measurements over time of both samples after resuspension in TMAOH 1 mM and at the same NP concentration.

At the source of these discrepancies, one can observe the diversity of the materials actually employed in the different studies, which are presented under the same name. For instance, most research regarding CeO_2_ NP toxicity has been performed with commercially available NPs (often supplied in dry aggregated form) in order to assess the consequences of occupational and environmental exposure. These are different materials from those produced by wet chemistry routes in the laboratory, where the NPs are always kept isolated and well dispersed. In addition, for these types of studies, administered doses are usually higher than those proposed in nanomedicine (Figures 1A,B). In addition to their different initial characteristics, these materials are often prone to aggregation when dispersed into biological fluids, such as complete cell culture medium or serum (5, 39). For instance, He et al. (39) showed how intratracheally instilled CeO_2_ NPs into Wistar rats agglomerate and form sediments in the bronchoalveolar medium. Consequently, the actual objects that cells encounter may behave very differently from the initially designed and produced NPs (Figure 1C).

**Figure 2:**
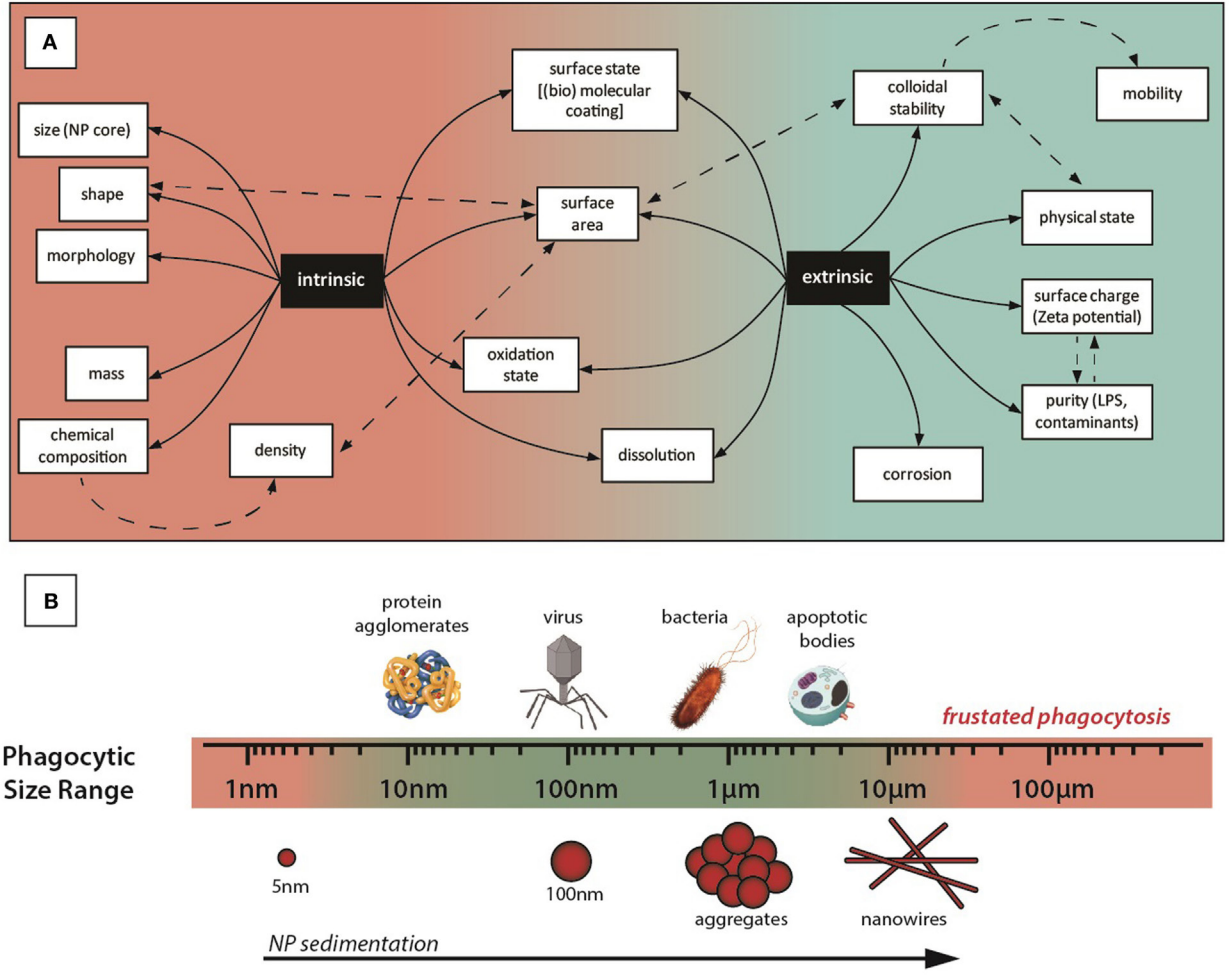
**(A)** Intrinsic and extrinsic properties of nanoparticles (NPs). Different properties of the NP, related to the NP itself (intrinsic) or to the NP behavior in the exposure media (extrinsic). For instance, we can design CeO_2_ NPs with specific sizes and shapes, but agglomeration in the exposure media leads to specific surfaces, concentrations, mobilities, etc., very different from the initially prepared NPs. As agglomerated NPs behave as a large particle, this makes the NP more immunogenic and affects the concentration of NPs in different parts of the body, where they are accumulated in organs of the MPS system. Importantly, for the (immuno)toxicity aspects, agglomerates of NPs are no longer on the nanometric regime of sizes and may have similar consequences as the incidental inorganic microparticles, extensively investigated during the last century: burning oil residues, silica from mining or asbestos have been found stacked in affected tissues, causing pathologies such as silicosis, asbestosis, and/or inflammatory reactions. Thus, in this example, even if CeO_2_ NPs are not toxic (and therapeutically beneficial) by themselves, they may be risky because they could be a source of toxic aggregates. **(B)** Graphical representative sizes of key entities capable of generating immune response and different NP morphologies and NP aggregates.

Comparing studies regarding nanomedicine and nanosafety, it seems that often the differently observed biological effects of NPs are related not only to its parental composition and purity but also to its final aggregation state (40), which is independent of the employed material and can be reproduced with other NPs. For instance, aggregates of TiO_2_ (41), Al_2_O_3_ (42), and Fe_2_O_3_ (43) NPs show similar toxicity to CeO_2_ aggregates (37, 44), as well as CeO_2_ (45) or Au NPs (46) carrying cationic amphipathic molecules on their surfaces have been observed to be similarly toxic. Regarding aggregates, in the case of CeO_2_, Rogers et al. (44) evaluate how exposure to different concentrations of aggregated CeO_2_ NPs affects indices of whole animal stress and survivability in *Caenorhabditis elegans*. Results showed that CeO_2_ aggregates promoted strain-dependent decreases in animal fertility, a decline in stress resistance as measured by thermotolerance and shortened worm length. Moreover, chronic exposure of CeO_2_ NP aggregates was found to be associated with increased levels of ROS and heat shock stress response (HSP-4). Regarding surface state, Dowding et al. (45) prepared different samples of CeO_2_ NPs using identical precursor (Cerium nitrate hexahydrate) through similar wet chemical process but using different oxidizer/reducer: H_2_O_2_, NH_4_OH, or hexamethylenetetramine (HMT). Results showed that unlike the other CeO_2_ NPs preparations, HMT-CeO_2_ NPs were readily taken into endothelial cells and reduced cell viability at a 10-fold lower concentration than the others. This indicates that the biological effects of NPs depend not only on intrinsic but also extrinsic features, aspects related to the NP itself and to its history and environment. Thus, colloidal stability, which determines the agglomeration and sedimentation, depends on the concentration and nature of ions and molecules present in the media at a certain temperature. This affects the hydrodynamic radius, which depends on temperature and viscosity; NP corrosion, which depends on the combined redox potential of the species present in the environment; and speciation of leached ions, which depends on the nature of the dispersing media (Figure 2A). The NP concentration will affect the kinetics of the previously coexisting phenomena.

In this context, interactions between NPs and the immune system are of particular interest for both their efficient use and their safety in biomedical applications. NPs are foreign objects, sized within the range of that detected and managed by the immune system, which has a responsibility for categorizing invasion and providing an appropriate response (Figure 2B). For example, NPs may exacerbate immune responses by ordering and repetition of ligands (47–49), as well as by altering redox status, both increasing (50) and decreasing ROS and inflammatory mediator levels (14).

## The Apparent Contradiction

Lack of understanding NP characteristics and their evolution inside biological media is recognized as one of the key points underpinning the abovementioned controversies (40). Thus, as with many other inorganic NPs employed in nanomedical research, CeO_2_ NPs evolve when in contact with physiological media (5, 51). This evolution may entail the loss of intended catalytic activity, transforming beneficial NPs into deleterious ones. The most significant alterations affecting the biological fate and effects of NPs when dispersed in biological media are: (i) agglomeration and aggregation of the NPs (5, 52, 53), (ii) formation of the NP protein corona as a result of the adsorption of proteins onto the inorganic surface (54, 55), and (iii) NP corrosion and/or dissolution into ionic species (56–59). Indeed, it has been proposed that the higher toxicity of unstable preparations of NPs may not be due to the material *per se* but to its rapid aggregation into final micro- or macrometric sizes (5, 51) and the leaching of toxic ionic species into the solution (57). For instance, in the work of Kirchner et al. (57), the release of toxic Cd^2+^ ions from CdSe and CdSe/ZnS NPs and their stability toward aggregation were demonstrated to play an important role for the observed cytotoxic effects. Similarly, aggregation of NPs has been shown to clearly determine the exposure of NPs to cells. Xia et al. (50), comparing the toxicity induced by different ambient and manufactured NPs, showed a dramatic change in their state of aggregation, dispersibility, and charge during transfer from a buffered aqueous solution to cell culture medium and how it affects the observed cellular responses. Cho et al., (60) studied how sedimentation affected the cellular uptake of gold NPs in *in vitro* experiments, dramatically altering their exposure and biological effects. Typically, *in vitro* experiments measure the uptake of NPs by exposing cells at the bottom of a culture plate to a suspension of NPs, and it is generally assumed that the suspension is well dispersed. But, if NPs sediment, their concentration on the cell surface may be higher than the initial bulk concentration, and this could lead to increased uptake by cells. Indeed, results showed that cellular uptake of gold NPs mostly depended on the sedimentation and the diffusion velocities of the NPs.

Other NP transformations can also alter biological responses, leading to unexpected results. For example, Xue et al. (61) reported that CeO_2_ NPs can protect DNA from damage in Tris–HCl and sulfate buffers, but not in phosphate-buffered saline. A mechanism of action was proposed: cerium phosphate is formed on the surface of the NPs, which interferes with redox cycling between Ce^3+^ and Ce^4+^. As a result, the antioxidant activity of CeO_2_ NPs is greatly affected by the external environment. Similarly, Perez et al. (62) observed that the antioxidant properties of CeO_2_ NPs were pH-dependent. They suggested that a high concentration of H^+^ interferes with the regeneration of Ce^3+^, resulting in a loss of antioxidant activity. However, disintegration of CeO_2_ in acidic media could also account for the observed effects, similar to NP disintegration observed in different media (57, 63).

Given these effects, when conducting studies involving NPs for safety or medicine, it is essential to understand the changes that take place with their insertion into biological media, from complete cell culture media, to full blood, or lymph, to the intracellular cytoplasm. This includes NP colloidal stability, vicinity interactions, chemical transformations, association with plasma proteins, interaction with components of the immune system, and traditional absorption, distribution, metabolism, and excretion studies adapted to the unique specifications of NPs. Additionally, NPs can be complex and composed of different entities, all of which can have different fates. As an example, in the work of Feliu et al. (64), the authors review a vast collection of recent scientific literature indicating that NPs *in vivo* should no longer be considered as homogeneous entities. They conceptually divide a NP into the inorganic core, the engineered surface coating, comprising of the ligand shell and optionally also bio-conjugates, and the corona of adsorbed biological molecules. The authors found empirical evidence showing that all of these three described components may degrade individually *in vivo*. Due to this, the life cycle and biodistribution of the whole heterostructure is drastically modified.

## Concluding Remarks

There is an increasing number of conflicting reports on the impact of CeO_2_ NPs on oxidative stress and inflammation, with some studies reporting the promotion of oxidative stress induced by immune system activation, and others reporting protective effects against inflammatory processes. To overcome this apparent contradiction, understanding the physicochemical transformations and evolution of the NPs in biological systems is imperative. Understanding these mechanisms will enable the design of nanomaterials that work more precisely in medicine and safely in society.

The majority of negative immune effects reported in the scientific literature are related to NP aggregation and contamination, which cause biological effects independent of the composition, size, and shape of individual NPs. Generally, isolated, non-contaminated NPs show no toxicity, while contaminated and aggregated NPs are often described as immunotoxic (65, 66). This is especially dramatic in the case of CeO_2_ NPs, which have been reported many times as anti-inflammatory or pro-inflammatory, often without a proper description of the material used or its purity (40).

## Author Contributions

All authors listed have made substantial, direct, and intellectual contributions to the work and approved it for publication. VP suggested the topic and provided the concept and design of the work. EC and VP retrieved the relevant literature, compiled all information on the topic, and wrote the mini-review. MG contributed to the design of graphical information. JP contributed to the cerium oxide reactivity sections, retrieving the relevant literature, and participating in the discussion and writing. GC and WJ contributed to the oxidative stress, inflammatory processes, and antioxidant activity sections by retrieving the literature and participating in the discussion and writing.

## Conflict of Interest Statement

The authors declare that the research was conducted in the absence of any commercial or financial relationships that could be construed as a potential conflict of interest.
