# Corrigendum: Intrinsic and Extrinsic Properties Affecting Innate Immune Responses to Nanoparticles: The Case of Cerium Oxide

**DOI:** 10.3389/fimmu.2017.01891

**Published:** 2017-12-22

**Authors:** Eudald Casals, Muriel F. Gusta, Jordi Piella, Gregori Casals, Wladimiro Jiménez, Victor Puntes

**Affiliations:** ^1^Vall d’Hebron Institut of Research (VHIR), Barcelona, Spain; ^2^Institut Català de Nanociència i Nanotecnologia (ICN2), CSIC, The Barcelona Institute of Science and Technology (BIST), Campus UAB, Barcelona, Spain; ^3^Biochemistry and Molecular Genetics Service, Hospital Clínic Universitari, IDIBAPS, CIBERehd, Barcelona, Spain; ^4^Department of Biomedicine, University of Barcelona, Barcelona, Spain; ^5^Institució Catalana de Recerca i Estudis Avançats (ICREA), Barcelona, Spain

**Keywords:** nanoparticles, cerium oxide, nanoparticle evolution, nanoparticle agglomeration, ion leaching, antioxidant activity, inflammation, immune response

In the original article, we overlooked to include the FutureNanoNeeds Project financed by the European Community under the FP7 Programme under Grant Agreement No. 604602 (FP7-NMP-2013-LARGE-7, Grant Agreement No. 604602) to Victor Puntes. The authors apologize for this error and state that this does not change the scientific conclusions of the article in any way.

A correction has been made to the text in the Funding: “Financial support from the FutureNanoNeeds Project (GA: 604602) financed by the European Community under the FP7 Programme (FP7-NMP-2013-LARGE-7) is gratefully acknowledged.”

The original article was updated.

## Conflict of Interest Statement

The authors declare that the research was conducted in the absence of any commercial or financial relationships that could be construed as a potential conflict of interest.

